# Supplementary material for: Protein Kinase A Catalytic and Regulatory Subunits Interact Differently in Various Areas of Mouse Brain
Source: Int J Mol Sci. 2020 Apr 26;21(9):3051. doi: 10.3390/ijms21093051 (PMC7246855; doi:10.3390/ijms21093051)
Supplement: Supplementary file 1 [file ijms-21-03051-s001.pdf]

Protein Kinase A catalytic and regulatory subunits interact differently in various areas of mouse brain

C. Mucignat-Caretta and A. Caretta

SUPPLEMENTARY MATERIAL

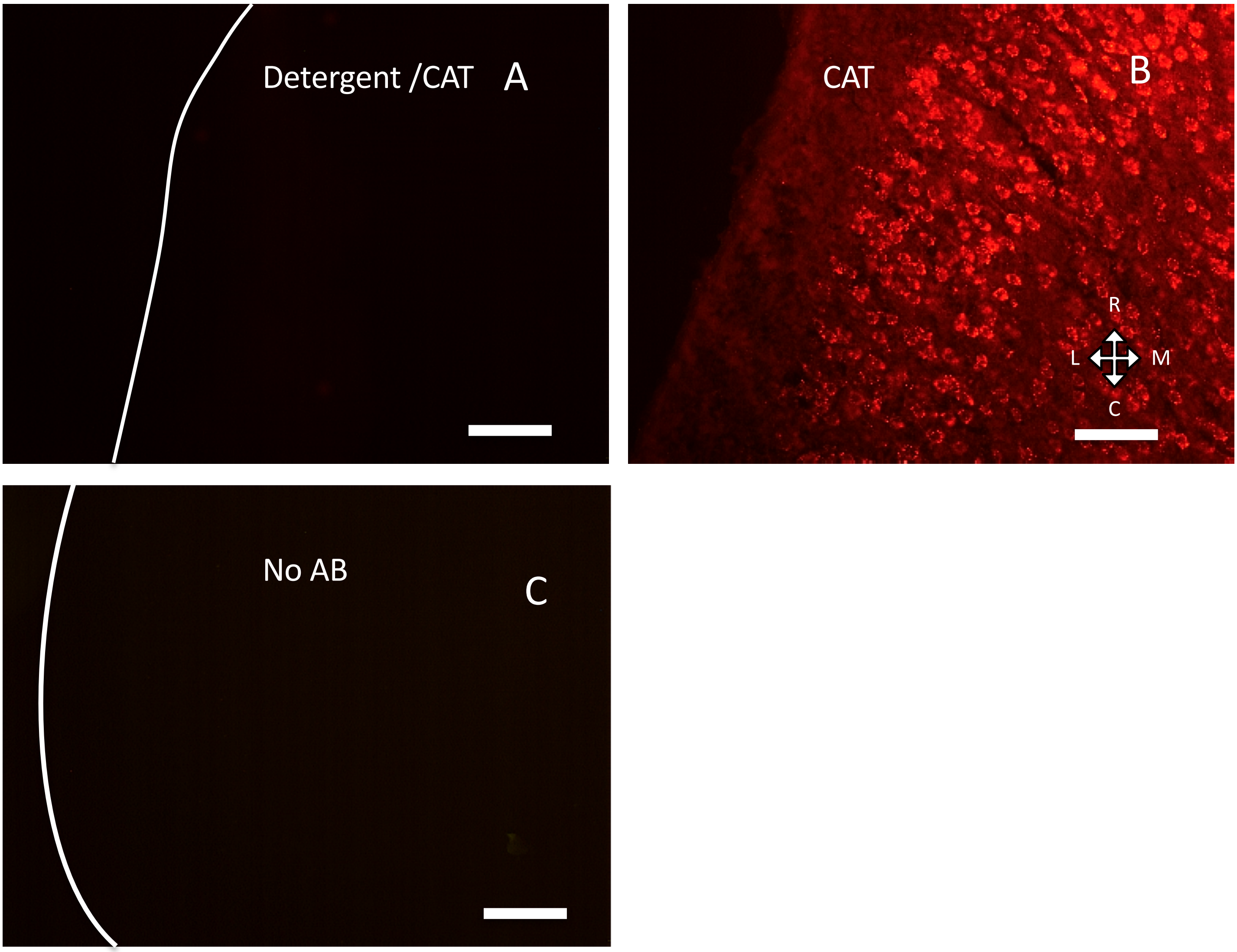

Figure S1. PKA catalytic subunit immunolabelling of the parietal S1BF cerebral cortex under different conditions, unmodified images. Horizontal sections, scale bar: 50 μm. The white lines in A and C outline the pial surface. (**A**) no labelling with anti-catalytic subunit (CAT) antibody is apparent because of pre-treatment of the section with detergent (Triton X-100 1% in PBS for 20 minutes) before fixation in formalin. (**B**) in the subsequent section of parietal S1BF cortex, the same area is immunolabelled by anti-catalytic subunit (CAT) antibody (see Materials and methods for fixation and immunofluorescence conditions). Images **A** and **B** are obtained from the same experiment, using the same parameters for image acquisition. (**C**) no labelling is apparent if the primary antibody is omitted (No AB); no autofluorescence is present.

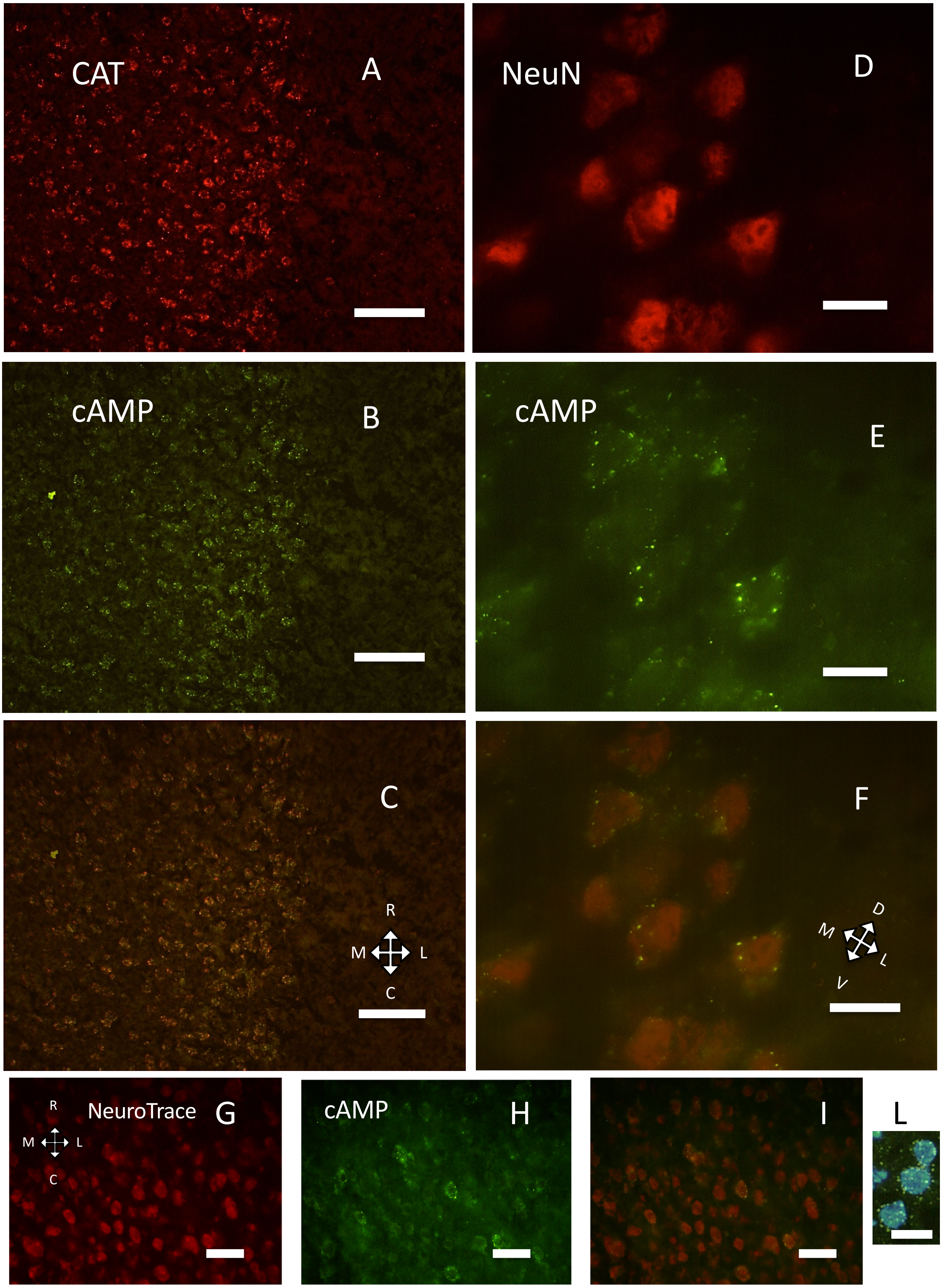

Figure S2. Parietal S1BF cortex horizontal sections. (**A**) at lower magnification compared to **Fig. 1 (A-F)**, PKA catalytic immunolabelling can be appreciated in all strata of the parietal cortex. Pia on the right. (**B**) SAF-cAMP in the same field as **A**. (**C**) merge of **A** and **B**, showing coincidence of the two signals. (**D**) NeuN immunolabelling of the parietal cortex. (**E**) Fluorescent cAMP (SAF-cAMP) in the same field as **D**. (**F**) merge of **D** and **E**, showing that cAMP binds in proximity of the neuronal nuclei marker. (**G**) NeuroTrace labelling in S1BF cortex. (**H**) Fluorescent cAMP (SAF-cAMP) labelling of the same field as **G**. (**I**) merge of **G** and **H**. (**L**) DAPI counterstaining confirms perinuclear localization of fluorescent SAF-cAMP. Scale bar: 10 μm (**D-F**, **G-L**), 50 μm (**A-C**).

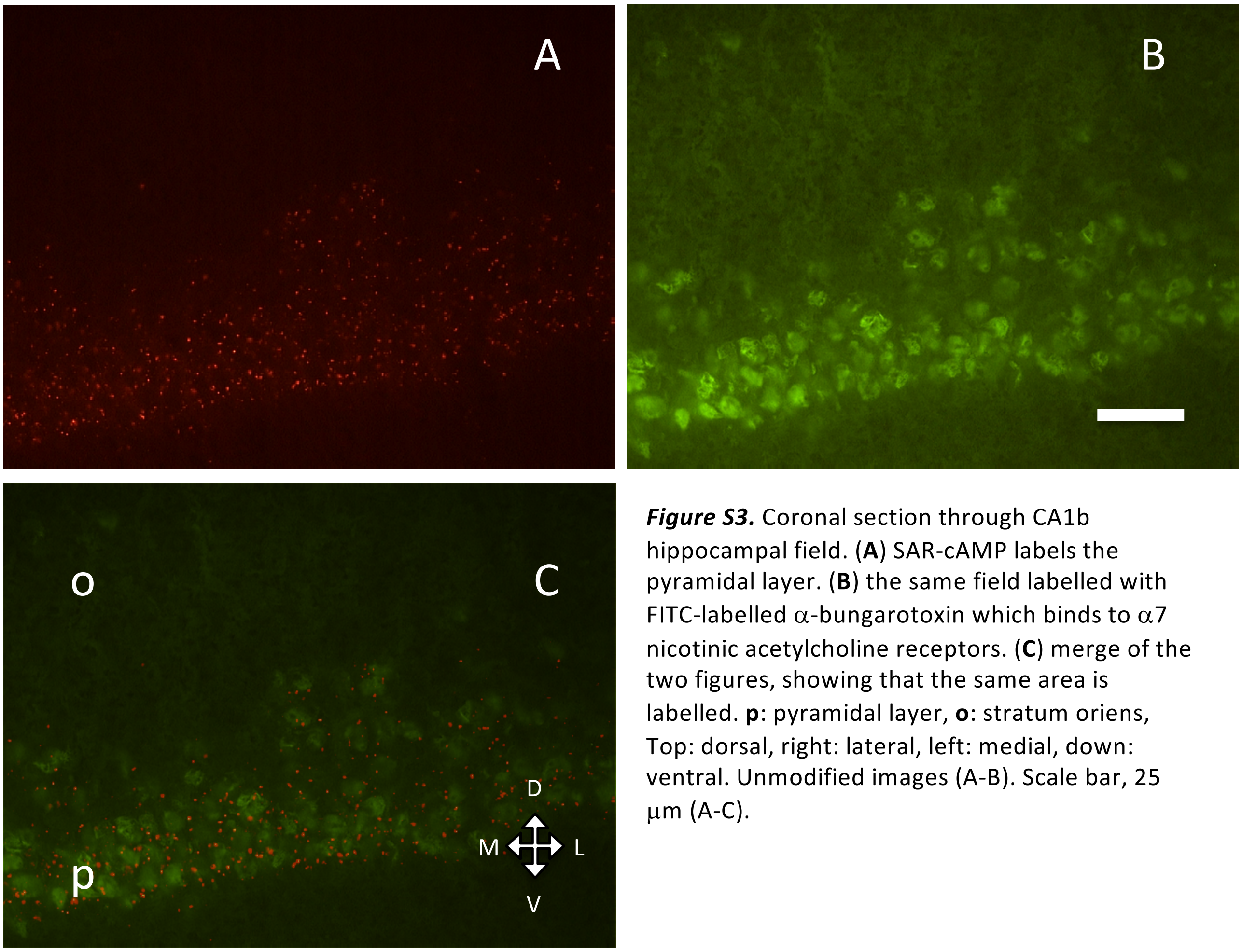

Figure S3. Coronal section through CA1b hippocampal field. (**A**) SAR-cAMP labels the pyramidal layer. (**B**) the same field labelled with FITC-labelled α-bungarotoxin which binds to α7 nicotinic acetylcholine receptors. (**C**) merge of the two figures, showing that the same area is labelled. **p**: pyramidal layer, **o**: stratum oriens, Top: dorsal, right: lateral, left: medial, down: ventral. Unmodified images (A-B). Scale bar, 25 μm (A-C).

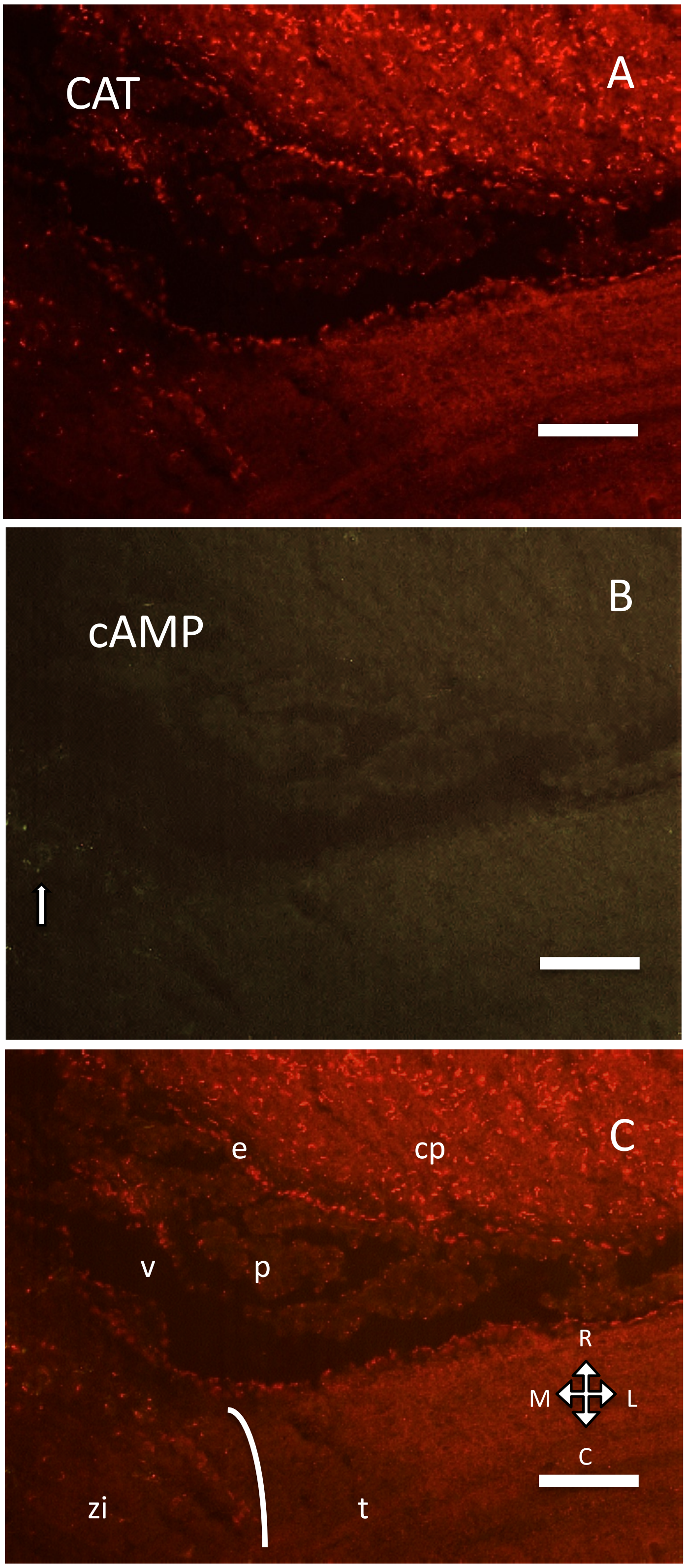

Figure S4. Horizontal section through the lateral ventricle. (**A**) PKA catalytic subunit differently labels various structures in the brain. (**B**) the same area is almost unlabeled by Alexa488-cAMP, except for a handful, very faint dots in the left part of the figure (arrow), corresponding to the zona incerta, while the cerebral cortex is heavily labeled, in the same section (outside this field, not shown). (**C**) merge of the two figures. Unmodified images. **cp**: caudate putamen, e: ependymal layer, **p**: choroideal plexi, **t**: posteromedial intralaminar thalamic nuclei, **v**: lateral ventricle, **zi**: zona incerta. Top: rostral, right: lateral, left: medial, down: caudal. Scale bar, 50 µm (A-C), 25 mm (D-F).

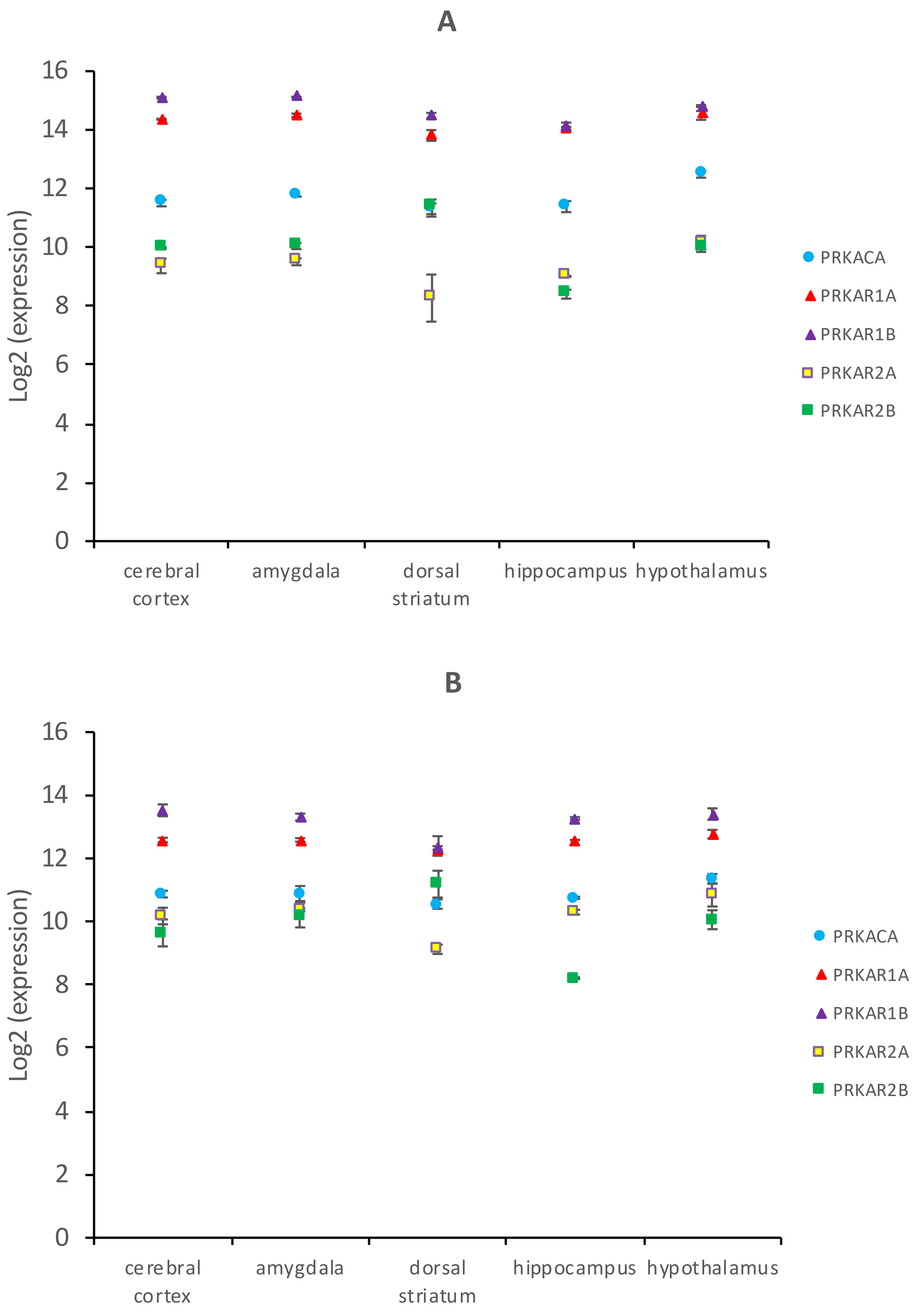

Figure S5. Expression levels of PKA catalytic and regulatory subunits in the mouse brain, obtained with microarray technology, as reported by two different data bases. Affymetryx probesetID: 1447720_x_at (PRKACA), 1452032_at (PRKAR1A), 1434325_x_at (PRKAR1B), 1452915_at (PRKAR2A), 1456475_s_at (PRKAR2B). Similar brain areas were selected. **A**: data accessed on BioGPS. **B**: data analysed with BrainStars. No link is apparent between protein expression and distribution in the different brain areas.

Figure S6 Part 1 (A-H).

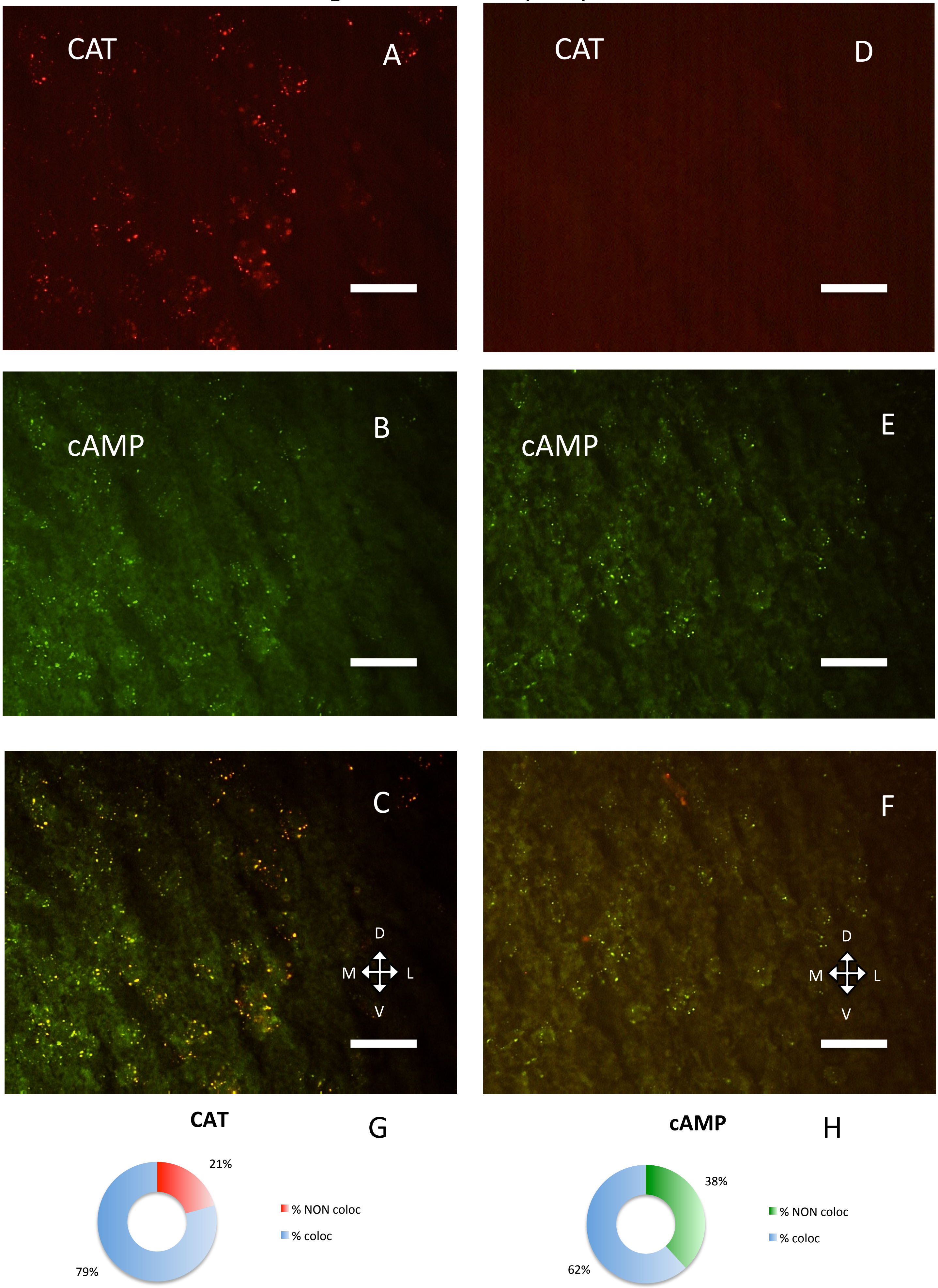

Figure S6 Part 2 (I-N).

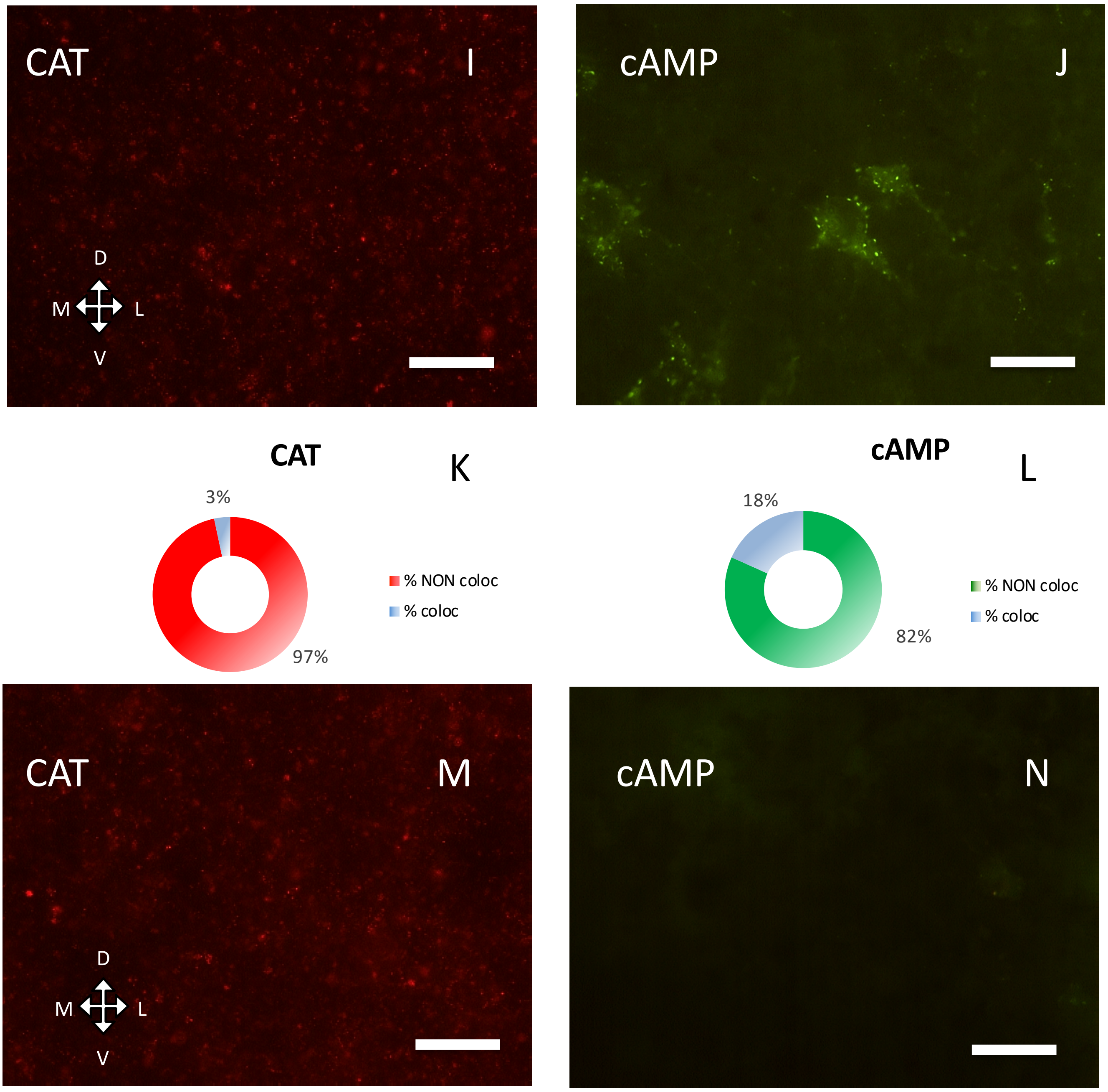

FIGURE S6. Effect of cAMP on PKA catalytic subunit in the cerebral cortex. Coronal sections, unaltered images. (**A**) in control conditions, PKA catalytic subunit immunolabelling of the cerebral S1BF cortex, pia on the lower right. (**B**) same field, fluorescent SAF-cAMP. (**C**) merge of **A** and **B**, showing coincidence of the signals. (**D**) after cAMP addition, no PKA catalytic immunolabelling is apparent, however at higher magnification (see main text, **Fig. 6 C**) a punctuated signal can be appreciated in S1BF cortex. Pia on the lower right. (**E**) in the same field SAF-cAMP still binds to regulatory subunits, being the localization of binding sites for cAMP apparently unaffected. (**F**) merge of **D** and **E**. **G-H**: quantification of superimposition in F (n=1262). (**G**) Percentage of PKA catalytic immunolabelling colocalizing (% coloc, light blue, n=408) or not (% NON coloc, red, n=106) with fluorescent cAMP in **C**. (**H**) Percentage of fluorescent cAMP colocalizing (% coloc, light blue, n=462) or not (% NON coloc, green, n=286) with PKA catalytic immunolabelling in **C**. (**I**) In the olfactory cortex, cAMP induces relocalization of catalytic subunit, while in the same field (**J**) Alexa488-cAMP labelling is apparent in neurons. **K-L**: quantification of superimposition in I and J (n=2596, merge image not shown). (**K**) Percentage of PKA catalytic immunolabelling colocalizing (% coloc, light blue, n=75) or not (% NON coloc, red, n=2171) with fluorescent cAMP. (**L**) Percentage of fluorescent cAMP colocalizing (% coloc, light blue, n=64) or not (% NON coloc, green, n=286) with PKA catalytic immunolabelling. (**M**) relocalization of PKA catalytic subunit in olfactory cortex. (**N**) same field, Alexa488-cAMP does not bind to regulatory binding sites after incubation with 8-Br-cAMP.
Scale bar, 25 μm (**A-F**), 10 μm (**I,J,M,N**).
